# 基于气相色谱-静电场轨道阱高分辨质谱的海参中风险物质的筛选

**DOI:** 10.3724/SP.J.1123.2022.04001

**Published:** 2022-10-08

**Authors:** Chang MA, Rong CAO, Shuai SUN, Haijun ZHANG, Jiping CHEN, Zhili XIONG, Xianbo LU

**Affiliations:** 1.沈阳药科大学, 辽宁 沈阳 110000; 1. Shenyang Pharmaceutical University, Shenyang 110000, China; 2.中国科学院分离分析化学重点实验室, 中国科学院大连化学物理研究所, 辽宁 大连 116023; 2. CAS Key Laboratory of Separation Science for Analytical Chemistry, Dalian Institute of Chemical Physics, Chinese Academy of Sciences, Dalian 116023, China

**Keywords:** 气相色谱-静电场轨道阱高分辨质谱, QuEChERs, 非目标分析, 环境污染物, 海参, gas chromatography-orbitrap high-resolution mass spectrometry (GC-Orbitrap HRMS), QuEChERs, nontarget analysis, environmental pollutants, sea cucumber

## Abstract

建立了一种基于改良QuEChERs的样品前处理新方法,并通过气相色谱-静电场轨道阱高分辨质谱(GC-Orbitrap HRMS)对海参样品中的有机氯农药类(OCPs)、多环芳烃类(PAHs)、酞酸酯类(PAEs)、多氯联苯类(PCBs)及其他农药类(ACs)等10大类289种具有健康风险的有机污染物进行了高通量高精准的定性鉴别和定量分析。通过将传统的QuEChERs方法与柱净化方法结合,提出了一种基于改良QuEChERs的简便生物样品制备新方法,经弗洛里硅土(Florisil)柱净化后脂含量降低了99.9%,显著减少了基质效应对分析结果的干扰,可在高分辨质谱(6万分辨率)全扫描模式下对289种目标化合物同时进行高精准定性筛查和定量分析。该方法定量限约0.56~57.8 pg/g,线性范围约6个数量级,回收率范围为40%~120%。由于Q Exactive GC-Orbitrap HRMS具备较高的质量分辨率和灵敏度,因此对目标化合物的定量限显著优于常规的色谱和质谱分析方法,常规方法无法检出的超痕量有机污染物利用该研究开发的方法可进行精准定量。基于此高覆盖度多目标分析方法对养殖场采集的海参样品进行分析,共从海参体内检出100种有机污染物,其中PAHs、ACs、PAEs、OCPs类检出总含量相对高于其他类污染物,总含量均值分别为157.8、153.2、64.4和46.4 ng/g dw。9-氯芴、5-氯苊、3-甲基胆蒽等多种新污染物首次在海参样品中检出,但含量都非常低。该方法简单高效,定量限低,线性范围宽,结果准确。此高覆盖度多目标分析方法可广泛应用于各种水产品中风险物质的广谱筛查和精准定量,为食品安全和绿色养殖提供技术支撑。

## Abstract (en)

A new method for sample pretreatment using improved QuEChERs was established, and 289 organic pollutants with health risks could be identified and quantified through gas chromatography-orbitrap high-resolution mass spectrometry (GC-Orbitrap HRMS). A high-resolution database of 289 environmental pollutants belonging to ten categories, including organochlorine pesticides (OCPs), polycyclic aromatic hydrocarbons (PAHs), phthalates (PAEs), polychlorinated biphenyls (PCBs), and other agricultural chemicals (ACs), was established for the non-targeted screening and quantitative analysis. A simple method for biological sample preparation using improved QuEChERs was proposed by combining a conventional QuEChERs method and a column purification method. After purification using a Florisil column, the lipid content was reduced by 99.9%, which significantly reduced the interference of the matrix effect observed during the analysis. Furthermore, simultaneous high-accuracy qualitative screening and quantitative analysis of the target compounds were performed through high-resolution mass spectrometry (60000 resolution) conducted in the full scan mode. The limits of quantification were 0.56-57.8 pg/g, presenting a large linear range (~10^6^), and the recovery range was 40%-120%. Due to the high-resolution and sensitivity of Q Exactive GC-Orbitrap HRMS, the limits of quantification of the target compounds were significantly lower than those achieved through methods based on conventional chromatography and mass spectrometry. Moreover, ultratrace organic contaminants that cannot be detected by conventional methods can be accurately quantified by the proposed method. Sea cucumber samples collected at the breeding site were analyzed using the proposed high-coverage multi-objective analytical method, and more than 100 types of organic pollutants were detected; the mean contents of PAHs, ACs, PAEs, and OCPs were 157.8, 153.2, 64.4, and 46.4 ng/g dw, respectively, which were higher than those of other pollutants. Some new contaminants, such as 9-chlorofluorene, 5-chloroacenaphthene, and 3-methylcholanthrene, were detected at very low contents for the first time in the sea cucumber samples. The proposed method is simple and efficient, allows the detection of pollutants at very low contents, and provides accurate and reliable results. Thus, this high-coverage multi-objective analytical method can be widely used for broad-spectrum screening and accurate quantification of contaminants in various aquatic products, providing technical support for food safety control.

全世界使用的化学品数量正在稳步增加,新化合物还在不断合成。因此,在环境领域经常能发现各种新污染物^[1]^。化学品使用量的增加引起了科学家和决策者对环境污染的担忧^[2]^。一些人为污染物,如农药、工业副产品、药品和个人护理产品等被释放到环境中,可能对生物体的健康、生存和繁殖造成巨大威胁^[3]^,并通过动植物尤其是水产品的生物累积和饮食摄入等多种方式对人类健康造成潜在威胁。污染物通过各种方式在生物体内进行累积且不同污染物之间具有累积性差异,这些污染物在环境和生物体内通常处于痕量水平,因此污染物的痕量多残留检测技术开发为目前国内外的重点研究方向,特别是通过快速、高覆盖度、精准的分析方法实现痕量多残留检测成为研究的焦点^[4]^。目前痕量污染物多残留检测通常采用气相色谱-质谱、液相色谱-质谱等方法,其中静电场轨道阱高分辨质谱(Orbitrap HRMS)的高分辨全扫描模式可同时采集所有目标化合物信息,可对复杂基质中大量污染物进行广谱筛查、定性鉴别并同时对多种目标化合物进行定量分析。Q Exactive GC-Orbitrap HRMS系统不仅具有气相色谱-三重四极杆质谱的定量能力,还具备只有Orbitrap HRMS技术才能提供的全扫描高分辨率、精确质量(HRAM)性能,因此面对靶向和非靶向分析均能提供高灵敏度,且对靶向分析可获得高置信度的定量结果。

海参(*Holothurian*, sea cucumber)属于无脊椎动物、棘皮动物门、海参纲。《本草纲目》记载:海参,味甘咸,补肾,益精髓、摄小便、其性温补,足敌人参,故名海参^[5]^。海参具有高蛋白、低脂肪、低糖等优点,富含各种人体必需的氨基酸、维生素、常量和微量元素以及多种重要的活性成分^[6⇓⇓⇓⇓⇓⇓-13]^。海参作为人体滋补的重要营养品,其累积污染物的多残留监测对于海参养殖产业的绿色发展和保护人体健康都非常重要,但是目前的研究都局限在对抗生素、重金属等常规污染物的监测^[14,15]^,对海参中风险物质的高通量多残留分析却鲜有报道。

针对水产食品中风险物质的广谱筛选与污染物防控方面的需求,本研究建立了一种简单、有效的改良QuEChERS样品制备新方法,结合GC-Orbitrap HRMS方法所具备的高分辨、对目标污染物近乎全覆盖的优势,建立了用于海参中289种风险物质高通量筛查及高精准定量分析新方法。基于持久性、毒性、生物累积性等特性筛选危害物清单,针对水产品和养殖环境中重点关注及对人体饮食摄入产生潜在的健康危害等方面综合考虑,建立了共包含10大类289种环境污染物的高分辨数据库,可用于非靶向目标物筛查和定量分析。基于建立的样品前处理和GC-Orbitrap高分辨新方法,对养殖场采集的海参中污染物进行了高通量筛选和分析。该方法可广泛应用于水产品中风险物质的非目标筛查和精准定量,为食品安全风险评估提供技术支撑。

## 1 实验部分

### 1.1 仪器、试剂与材料

Q Exactive GC-Orbitrap高分辨质谱仪(配备AI1310自动进样器和TRACE 1310气相色谱仪,赛默飞世尔科技公司);冷冻干燥机(美国Labconco公司); Milli-Q超纯水仪(Merck Millipore公司); KQ-250DE型数控超声清洗器(昆山市超声仪器有限公司); XW-80A涡旋混合器(上海精科实业有限公司);台式离心机D-37520 Osterode(德国Kendro公司产品); DC-12氮吹浓缩仪(上海安普公司); R-205旋转蒸发仪(瑞士Buchi公司)。

农残级二氯甲烷、正己烷、壬烷购自美国J. T. Baker公司;色谱级乙腈购自美国Sigma-Aldrich公司;色谱级乙酸乙酯、分析级无水硫酸钠和无水硫酸镁购自天津大茂化学试剂厂;农残级弗洛里硅土选择性吸附剂(粒径60~100 μm)购自上海阿拉丁生化科技股份有限公司;分析级氯化钠购自天津天大化学试剂厂;^13^C标记的多氯联苯类(PCBs)标准品,P48-W-ES混合标准品(包括^13^C_12_-PCB-77、^13^C_12_-PCB-81、^13^C_12_-PCB-105、^13^C_12_-PCB-114、^13^C_12_-PCB-18、^13^C_12_-PCB-123、^13^C_12_-PCB-126、^13^C_12_-PCB-156、^13^C_12_-PCB-157、^13^C_12_-PCB-167、^13^C_12_-PCB-169、^13^C_12_-PCB-189), P48-M-ES混合标准品(包括^13^C_12_-PCB-28、^13^C_12_-PCB-52、^13^C_12_-PCB-101、^13^C_12_-PCB-138、^13^C_12_-PCB-153、^13^C_12_-PCB-180), P48-RS混合标准品(包括^13^C_12_-PCB-70、^13^C_12_-PCB-111、^13^C_12_-PCB-170),多氯萘(PCN)-MXA混合标准品,PCN-MXC混合标准品,多溴联苯醚(PBDEs)混合标准品,多氯代二苯并对二噁英(PCDDs)与多氯代二苯并呋喃(PCDFs)标准品均购自加拿大Wellington Laboratories公司;PCBs标准品、有机氯农药类(OCPs)标准品、多环芳烃类(PAHs)标准品、氯苯类标准品、苯胺类(anilines)标准品、酚类(phenols)标准品、酞酸酯类(PAEs)标准品均购自美国Organic Standards Solutions International公司;氯代多环芳烃(Cl-PAHs): 5-氯二氢苊(5-ClAce, 纯度97.6%)、3,5-二氯苊(3,5-Cl_2_Ace, 纯度96%)、3,5,6-三氯苊(3,5,6-Cl_3_Ace, 纯度98.9%)、3,5,6,8-四氯苊(3,5,6,8-Cl_4_Ace, 纯度96%)、9-氯芴(9-ClFlu, 纯度96%)、9-氯菲(9-ClPhe, 纯度95%)固体标准品购自Toronto Research Chemicals, Inc.(Toronto, Canada); 9-氯蒽(9-ClAnt, 纯度96%)固体标准品购自Matrix Scientific (MA, USA); 2-氯蒽(2-ClAnt, 纯度99.9%)和9,10-二氯蒽(9,10-Cl_2_Ant, 99.5%)固体标准品购自Tokyo Chemical Industry (Tokyo, Japan); 1-氯芘(1-ClPyr, 纯度98%)标准品购自Fluorochem(Derbyshire, UK); 6-氯苯并[*a*]芘(6-ClBaP, 50 mg/L)标准溶液购自Chiron (Trondheim, Norway)。另外28种混合标准品均购自美国AccuStandard公司,50 mL离心管购自美国Corning公司。

### 1.2 标准溶液的配制

将所有Cl-PAHs的固体标准品以二氯甲烷溶剂配制成质量浓度为0.44~0.69 mg/mL的单一储备溶液。然后分别移取适当体积的Cl-PAHs单一储备溶液,以正己烷稀释成质量浓度为5.0 mg/L的混合储备溶液,待使用前移取适量体积稀释成所需浓度的标准溶液。PCBs回收率内标是将1000 μg/L的^13^C_12_-PCB-28、^13^C_12_-PCB-52、^13^C_12_-PCB-101、^13^C_12_-PCB-138、^13^C_12_-PCB-153、^13^C_12_-PCB-180用壬烷配制成质量浓度为100 μg/L的PCBs回收率内标。其他各种混合标准溶液均以壬烷稀释成质量浓度为100 mg/L的储备溶液,待使用前移取适量体积稀释成所需浓度的标准溶液,上述所有标准品、储备溶液及标准溶液均于-20 ℃冰箱中冷藏保存备用。

### 1.3 样本采集及样本前处理

样本采集 样本采集于辽宁省大连市普兰店区3个养殖点。新鲜海参样品经去离子水冲洗,去除内脏后获得可食用部分,通过食品粉碎机粉碎获得匀质样品。所有样品均经冷冻干燥后用粉碎机再次粉碎均匀,于-20 ℃密封保存待用。

样本前处理 样品提取与浓缩:准确称量1 g(±0.01 g)样品至50 mL离心管中,加入4 mL纯水并涡旋混匀1 min;快速添加10 mL乙腈-乙酸乙酯(1∶1, v/v)作为萃取溶剂,并涡旋混匀1 min后,加入4 g(±0.01 g)无水硫酸镁和1 g(±0.01g)氯化钠以去除水分并沉淀蛋白质。强烈涡旋混匀1 min后超声提取15 min, 3000 r/min条件下离心5 min,并收集上清液,重复上述提取步骤两次并合并上清液,通过旋转蒸发器将提取液浓缩至1~2 mL。

浓缩液净化 利用弗罗里硅土柱(Florisil柱,长度400 mm×内径20 mm)进行净化,填料从底层到顶层的添加顺序为:2 g无水硫酸钠、8 g弗罗里硅土、6 g无水硫酸钠,先用正己烷活化Florisil柱,然后将样品转移至该柱内,用100 mL正己烷-二氯甲烷(1∶1, v/v)混合溶液淋洗,将最后净化的洗脱液旋转蒸发至1~2 mL,逐步转移至微量管中在弱氮气流下吹干,并加入10 μL ^13^C标记的PCBs进样内标(100 μg/L)定容至10 μL。

### 1.4 色谱-质谱条件

TG-5SILMS色谱柱(30 m×0.25 mm×0.25 μm,购自赛默飞世尔科技);进样量为1 μL;以分流模式进样,分流比为5∶1,进样口温度260 ℃,流速1.0 mL/min;程序升温条件如下:初始温度40 ℃下保持1.5 min;以25 ℃/min的速率升温至90 ℃,保持1.5 min;再以25 ℃/min的速率升温至180 ℃;然后以5 ℃/min速率升温至280 ℃;最后以10 ℃/min速率升温至310 ℃后,保持10 min。

Q Exactive GC-Orbitrap HRMS使用电子轰击电离源(EI)和带高能碰撞诱导解离(HCD)的混合四极杆Orbitrap质谱仪进行操作。传输线温度为260 ℃,离子源温度为250 ℃,电子能量为70 eV。溶剂延迟时间为6.5 min,全扫描模式下,质谱扫描范围为*m/z* 50~750。氮气作为弯曲线性阱(C-Trap)缓冲、HCD碰撞气和仪器放空保护气,纯度为99.999%。质量分辨率设置为60000 FWHM(*m/z* 219),自动增益控制(AGC)设置为1×10^6^个离子,最大离子注入时间设置为“AUTO”。该仪器使用Xcalibur 4.1和TraceFinder 4.1(赛默飞世尔科技)进行控制。

### 1.5 数据处理

样品分析结果通过两种方式进行处理,其一是基于高分辨质谱数据库进行可疑物筛查与精准鉴别,其二是基于NIST数据库进行未知物的辅助定性。对未知物定性时设置筛查参数如下:至少存在2个匹配的精确质量数离子,质量准确度偏差小于5×10^-6^ (5 ppm),匹配得分大于80。对于NIST数据库的解析结果,通过匹配得分、保留指数、精确质量数偏差、质谱图、基峰与次基峰的比值等信息进行确证。本方法建立了包含多氯联苯、多氯萘、有机氯农药、多环芳烃等419种重点关注污染物的数据库子库,结合其他高分辨数据库,对化合物进行筛查。通过对混合标准品数据进行分析与处理后,可同时对289种化合物进行准确定量分析。

### 1.6 质量控制和方法性能

在每个测试序列开始之前使用全氟三丁胺(其碎片离子*m/z*分别为$CF_{3}^{+}$, 68.99466; $C_{3}F_{5}^{+}$, 130.99147; $C_{4}F_{9}^{+}$, 218.98508; C_8_F_16_N^+^, 413.97698; C_9_F_20_N^+^, 501.97059)进行外部质量校准,质量误差容限为±1 ppm(即±0.2 mDa);在测量期间,对毛细管色谱柱中流失的5个背景离子精确质量(C_2_H_3_OSi^+^, 73.04680; C_3_H_9_O_2_S $i_{2}^{+}$ , 133.01356; C_5_H_15_O_3_ ${\mathrm{Si}}_{3}^{+}$ , 207.03235; C_7_H_21_O_4_ ${\mathrm{Si}}_{4}^{+}$ , 281.05114; C_9_H_27_O_5_ ${\mathrm{Si}}_{5}^{+}$ , 355.06993)进行锁定,并设置搜索窗口为±2 ppm(±1 mDa),以对仪器进行内部质量轴校准。如果在某个扫描中,指定的背景离子中没有发现其确切质量±2 ppm,则不会对该扫描应用内部锁定。

采用本方法对289种污染物进行分析,包括18种PCBs(含12种二噁英类PCBs和6种指示性PCBs)、24种OCPs、16种PCNs、11种PAEs、37种PAHs、75种其他农药化学品类(ACs)、16种酚类化合物、34种苯胺类化合物、19种取代苯类以及39种药物、增塑剂等其他类毒性化合物(others)。本方法通过待测化合物与内标之间的相对响应因子对样品中的目标化合物进行定量分析。

## 2 结果与讨论

### 2.1 化合物数据库的构建

在本方法中,通过对标准物质进行全扫,获得所有必需的信息(保留时间、碎片离子、相对离子丰度等),从而构建数据库。由于混合标准品中包含多种目标化合物,可能产生大量相同相对分子质量不同结构的离子峰共流出,因此可通过正构烷烃对保留指数(RI)进行校准,通过解卷积插件对色谱图中可能存在的化合物进行区分。将获得的谱图与NIST标准谱库进行比较,通过碎片离子*m/z*及其分子式,进一步确定目标化合物及其保留时间和主要碎片离子。因为EI是一种高能电离模式,且仪器常在70 eV下进行电离,所以,最强的离子不完全为分子离子(只有一部分化合物以分子离子为基峰)。如果NIST谱库中不包含某些特定化合物,则可以通过手动修复主要碎片离子*m/z*来进行匹配。本工作通过关联目标化合物的保留时间、精确质量数等信息建立定性分析方法,并以内标法进行定量。

### 2.2 实验方法的优化

#### 2.2.1 提取方法的优化

传统QuEChERS方法利用溶剂对样品中的化合物进行提取,加入MgSO_4_等盐类除水后加入乙二胺-*N*-丙基硅烷(PSA)、C18键合硅胶(C18)等吸附剂去除杂质后对上清液进行仪器分析。而本方法针对提取溶剂及杂质净化方式进行了优化。QuEChERS方法中常用乙腈、乙酸乙酯、甲醇、丙酮等作为提取溶剂^[3,16⇓-18]^。本工作考察了不同萃取溶剂对目标化合物提取效果的影响,包括乙腈、乙腈-乙酸乙酯(1∶1, v/v)、丙酮-二氯甲烷(1∶1, v/v),并加入150 mg C18与150 mg PSA进行净化。为了避免样品本底值对回收率结果的影响,本实验以^13^C标记PCBs作为研究对象,^13^C-PCB-70、^13^C-PCB-111、^13^C-PCB-170作为内标考察各目标物的回收率,结果见图1。可以看出,乙腈-乙酸乙酯(1∶1, v/v)作为萃取溶剂时多数目标物回收率较高但是个别目标物回收率低,需要进一步优化QuEChERS前处理方法;而乙腈作为萃取剂回收率则较为稳定。

**图1:**
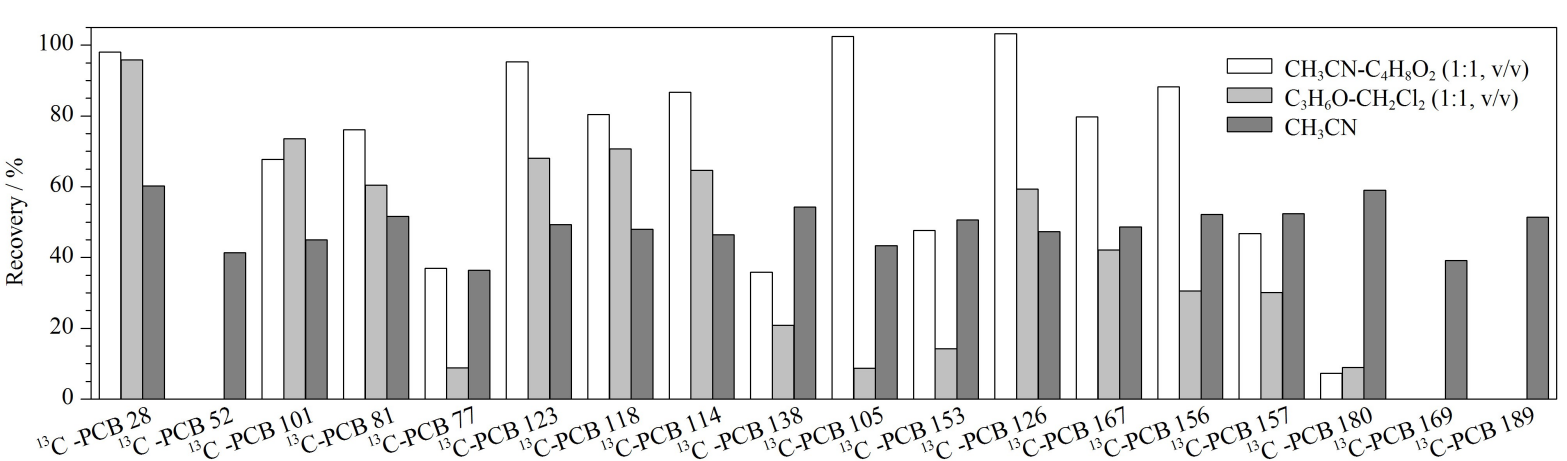
不同提取溶剂对^13^C-PCBs回收率的影响

#### 2.2.2 净化方法优化

经不同萃取剂提取浓缩后,管壁内均有黄色油状物质,表明150 mg C18与PSA作为净化剂并不能完全去除杂质;通过对该黄色油状物进行鉴定,证明该物质为胆固醇,由于其含量太高响应太大,严重影响其他目标物检测并会对仪器设备产生污染。在C18与PSA结合净化基础上,对样品中的胆固醇分别采用凝胶渗透色谱(GPC)、改性的聚苯乙烯/二乙烯苯高分子聚合物固相萃取填料(PEP)、氨基键合硅胶(Silica-NH_2_)、弗罗里硅土(Florisil)、氧化铝(Al_2_O_3_)等净化柱对其进一步净化,结果如图2所示。样品单独经C18+PSA进行净化后胆固醇峰面积高达1.08×10^12^(定义为100%),经Silica-NH_2_、GPC或PEP净化后胆固醇的峰面积分别降至C18+PSA净化方式的82.2%、56.3%、10.6%,证明上述3种方法对胆固醇均有一定净化效果但效果不理想,经Al_2_O_3_或Florisil净化后胆固醇峰面积响应值分别降至0.134%和0.129%,证明Al_2_O_3_与Florisil均具有非常理想的净化效果,经综合考量后选择Florisil作为净化材料。经乙腈、乙腈-乙酸乙酯(1∶1, v/v)分别提取后加入C18+PSA再结合Florisil进行净化,^13^C标记PCBs的回收率结果如图3所示,发现乙腈-乙酸乙酯(1∶1, v/v)的提取效果较乙腈提取效果更佳,因此后续采用乙腈-乙酸乙酯(1∶1, v/v)方法进行提取,并采用Florisil进行净化。

**图2:**
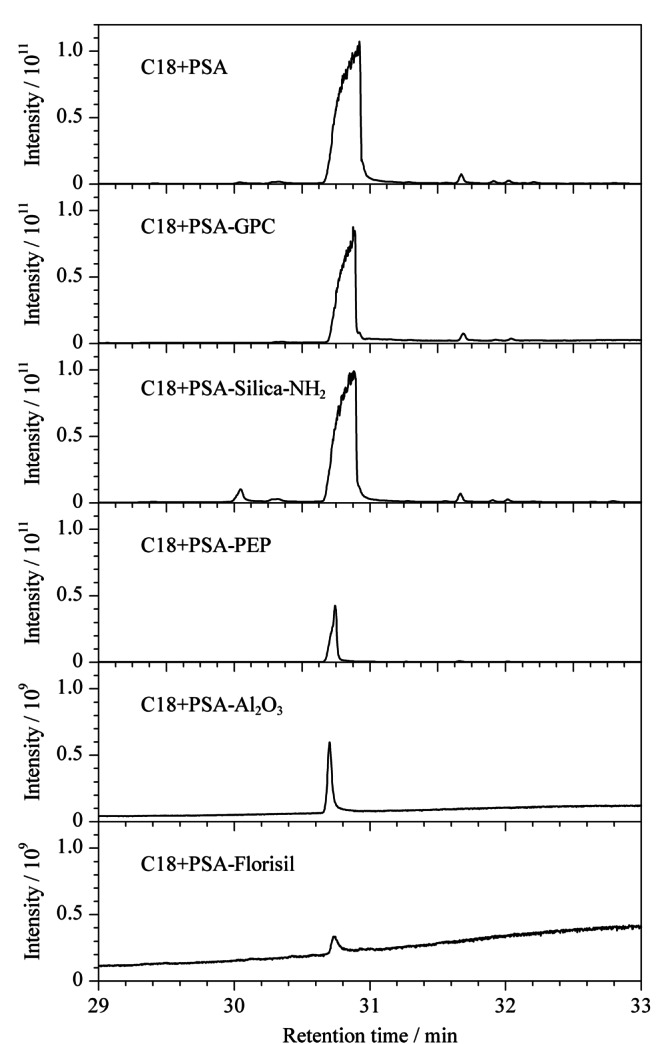
胆固醇通过不同净化手段在全扫描模式下的总离子流色谱图

**图3:**
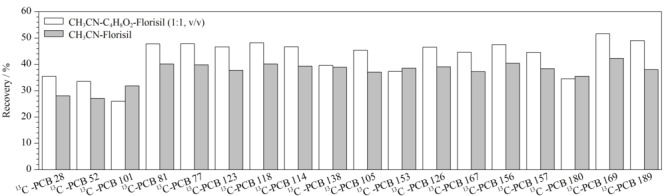
不同提取溶剂对^13^C-PCBs回收率的影响(经过Florisil柱净化)

由于C18、PSA与Florisil的净化效果存在叠加,C18与PSA对目标化合物有一定吸附效果导致回收率下降。因此后续样品前处理方法仅通过Florisil柱进行净化,不再使用C18与PSA。通过基质加标对回收率进行验证,结果显示依旧可以获得良好的净化效果且回收率可得到明显提高,PCBs回收率为67.8%~120%,所有目标物总回收率为40%~120%,综合考虑回收率可满足筛查分析的需求,且显著降低了基质效应对分析结果的干扰,因此最终选择只采用Florisil柱净化的方式进行样品前处理工作。

### 2.3 方法学考察

通过基质加标试验计算方法检出限,添加定量限附近浓度进行重复分析,以3倍标准偏差计算检出限,以3.3倍检出限作为定量限,最终检出限范围为0.17~17.5 pg/g,定量限范围为0.56~57.8 pg/g,线性范围约6个数量级。以基质加标试验对回收率进行考察,回收率范围为40%~120%,方法重复性RSD值为1.34%~24.5%。每6个样品添加一个程序空白进行质控,程序空白中的大部分目标污染物均低于检出限,仅个别目标物略高于检出限,在结果中需扣除空白干扰。

### 2.4 实际样品测定

利用Q Exactive GC-Orbitrap高分辨质谱对海参样品中289种污染物进行多目标同时分析。以二氯苯的3种同分异构体为例,图4a和4b分别为海参样品中与标准品(50 μg/L)的二氯苯总离子流色谱(TIC)图,图中同时叠加显示了1,2-二氯苯、1,3-二氯苯、1,4-二氯苯的一个定量离子(*m/z* 145.96848)与两个辅助离子(*m/z* 147.96549、110.99965)的离子流色谱图以及相应的离子丰度比,通过图4a和4b对比可精准确认这3种化合物为二氯苯。

利用以上方法对海参样本中10类有机污染物进行精准定性及定量分析,并对分析结果进行了统计,共从海参中检出100种污染物,其中包括7种PCBs、12种OCPs、3种PCNs、6种PAEs、27种PAHs、9种ACs、6种酚类化合物、7种苯胺类化合物、8种取代苯类化合物和15种其他类毒性化合物。各类有机污染物总含量均值分布情况如图5所示,其中PAHs、ACs检出含量较高,总含量均值分别为157.8 ng/g dw、153.2 ng/g dw,其次为PAEs、OCPs和其他类毒性化合物,总含量均值分别为64.4、46.4 和14.9 ng/g dw,其他5类有机污染物检出含量均小于1.6 ng/g dw,其中PCNs累计总含量均值仅为0.683 pg/g dw。

**图4:**
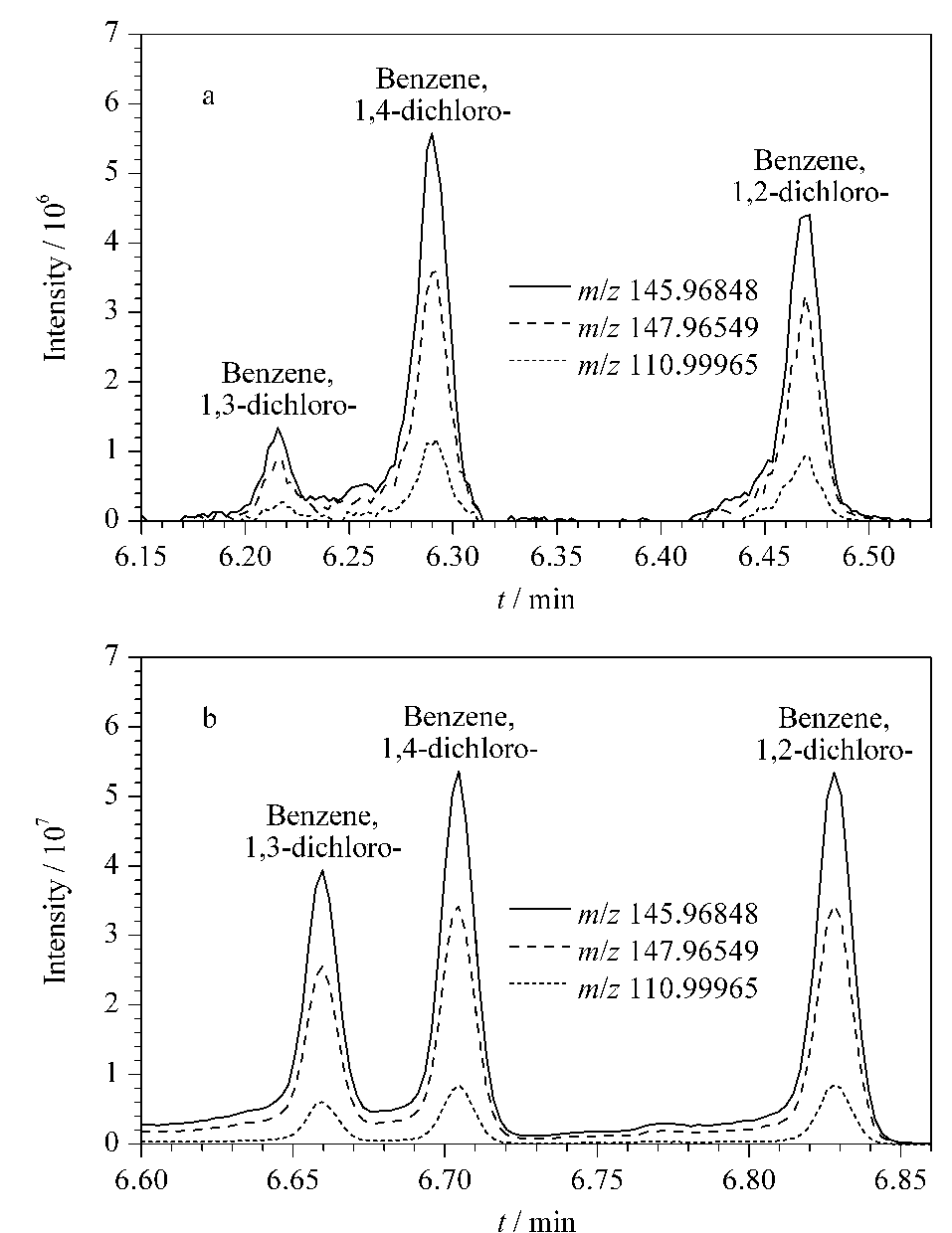
(a)海参样品中二氯苯与(b)二氯苯标准品的特征碎片离子分布

**图5:**
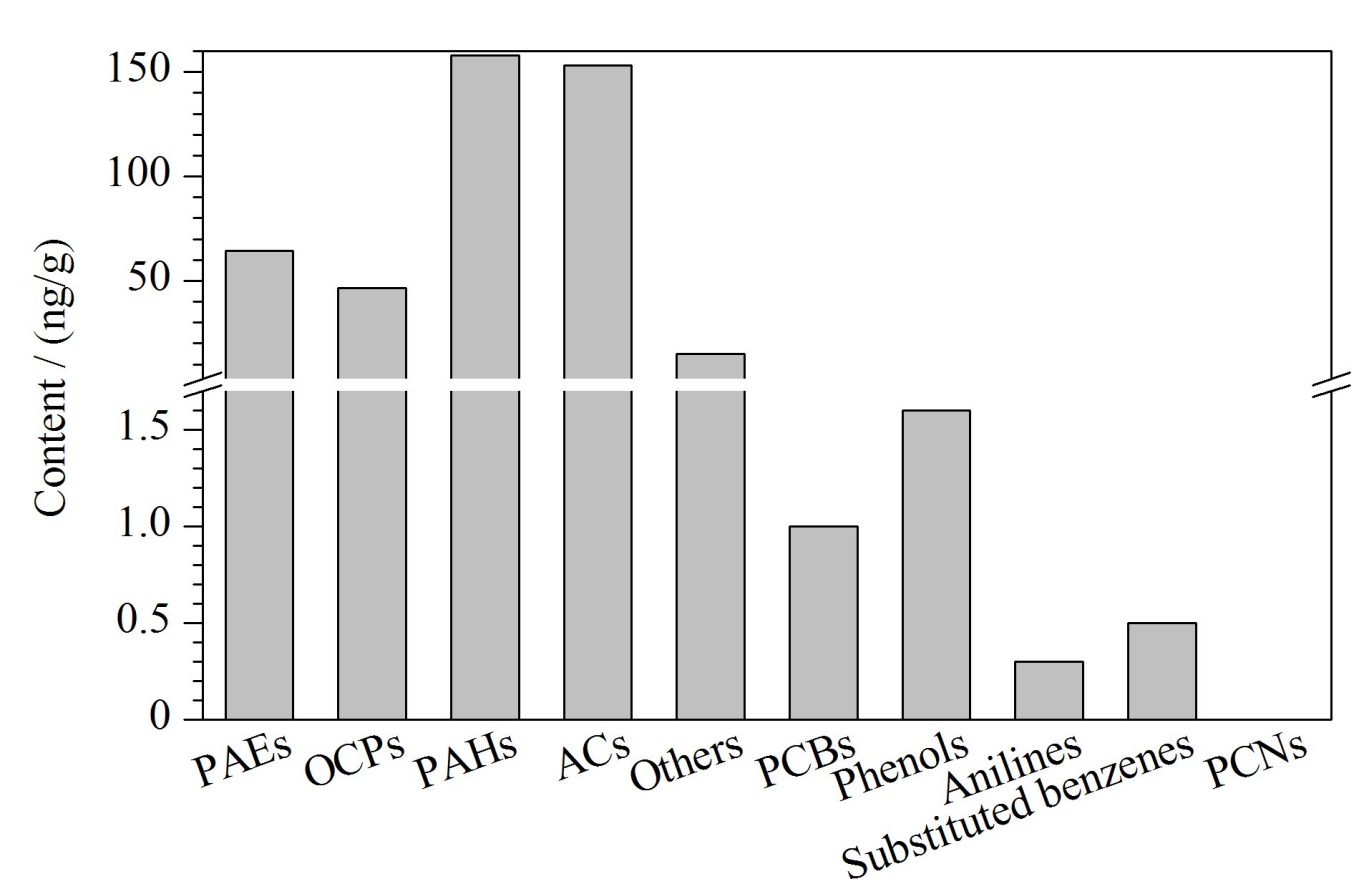
海参中各类有机污染物平均总含量分布图

除常见16种多环芳烃外,同时对海参中另外21种PAHs新污染物进行了分析,共分析了37种PAHs化合物,检出其中27种有机污染物,主要以3、4环形式存在,其中3环PAHs和4环PAHs分别占总PAHs的48.8%和43.6%。多种有机污染物首次在海参体内被检出,如9-氯芴、5-氯苊、3-甲基胆蒽等,平均含量分别为21.5 ng/g dw、1.09 ng/g dw、22.9 pg/g dw。对于检出的PCBs,主要以五氯和六氯取代贡献度较高,六氯取代如PCB-153其平均含量为0.365 ng/g dw,五氯取代如PCB-118其平均含量为0.279 ng/g dw,其他如PCB-52和PCB-167等检出含量较低,仅为1.28和3.99 pg/g dw。Leon等^[19]^对马尔梅诺环礁湖中海参的9种PCBs、14种PAHs等污染物进行了分析,PCBs含量为0.2~0.9 ng/g dw, PAHs含量为6.0~74.5 ng/g dw,经对比发现,马尔梅诺环礁湖海参中PCBs总量与普兰店海参样品中PCBs总量(平均含量0.99 ng/g dw)相当,普兰店海参中对应的14种PAHs总含量均值为127 ng/g dw,略高于马尔梅诺环礁湖中海参体内PAHs含量。

## 3 结论

本文结合传统的QuEChERs方法与柱净化方法,提出了一种基于改良QuEChERs的简便生物样品制备新方法,经弗洛里硅土柱净化后脂含量降低了99.9%,显著减少了基质效应对分析结果的干扰。依靠TraceFinder软件建立的高分辨数据库可在全扫描高分辨质谱模式下同时对289种目标化合物进行精准定性和定量分析。利用此方法对养殖场采集的海参中289种污染物分布进行分析,共检出100种有机污染物,其中PAHs、ACs、PAEs、OCPs检出总含量的均值相对高于其他类污染物。此外,9-氯芴、5-氯苊、3-甲基胆蒽等多种新污染物在海参中首次被发现,但含量相对较低。本方法具有样品前处理简单、灵敏度高、检出限低、线性范围宽、筛查分析精准等优势,具有很好的实际应用前景。该方法可广泛应用于水产品中风险物质的非目标筛查和精准定量,为食品安全风险评估提供有力的技术支撑。
